# Supplementary material for: Identification of photoperiod-induced specific miRNAs in the adrenal glands of Sunite sheep (Ovis aries)
Source: Front Vet Sci. 2022 Jul 22;9:888207. doi: 10.3389/fvets.2022.888207 (PMC9354845; doi:10.3389/fvets.2022.888207)
Supplement: Supplementary file 1 [file Data_Sheet_1.ZIP › Supplementary file/miRDeep2 file.zip!/miRDeep2 file/Know_Structure/LP42A3/oar-mir-544.pdf]

miRBase precursor : oar-mir-544
Total read count : 1032
oar-miR-544-5p read count : 983
oar-miR-544-3p read count : 41
remaining reads : 8

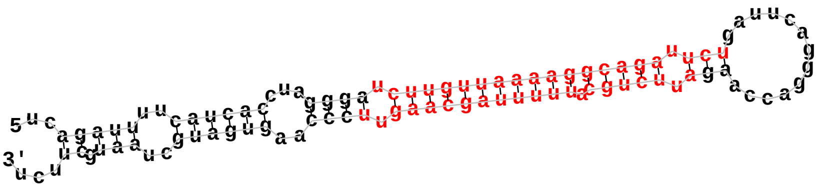

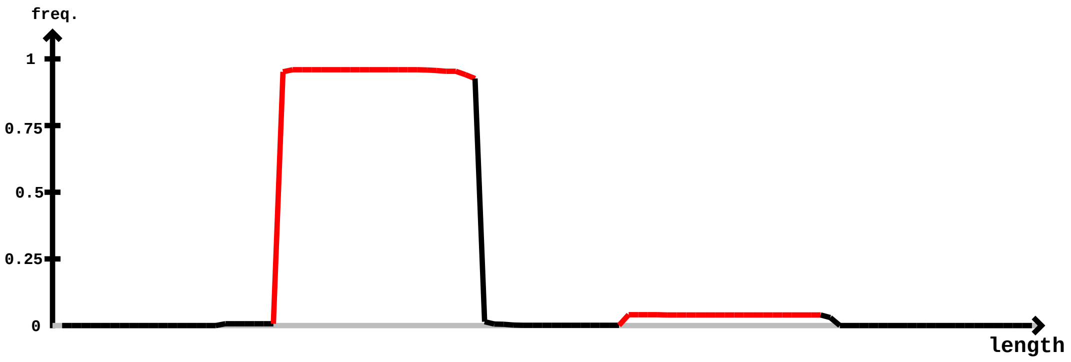


```
                                     oar-miR-544-5p
                                                                       oar-miR-544-3p
5'- ucagauuuucaucaccuagggaucuuguuaaaaggcagauucugauucagggaccaagauucugcauuuuuagcaaguucccaagugaugcuaaugcuucu   -3'    exp
    ..((((..((((((...((((.((((((((((((((((.(((.............))).))))).)))))))))))).))))..))))))..))).))...   reads     mm         sample
    ................uagggaucuuguuaaaaggca................................................................   1         0          seq
    ................uagggaucuuguuaaaaggcag...............................................................   2         0          seq
    ................uagggaucuuguuaaaaggcaga..............................................................   4         0          seq
    ......................ucuuguuaaaaggcagGuu............................................................   1         1          seq
    ......................ucuuguuaaaaggcagauA............................................................   2         1          seq
    ......................ucuuguuaaaaggcagauu............................................................   10        0          seq
    ......................ucuuguuaaaaggcagauuc...........................................................   15        0          seq
    ......................ucuuguuaaaaggcagauucu..........................................................   856       0          seq
    ......................ucuuguuaaUaggcagauucu..........................................................   1         1          seq
    ......................ucuuguuaaaaggcagGuucu..........................................................   1         1          seq
    ......................ucuuguuaaaaggcagauuUu..........................................................   2         1          seq
    ......................ucuuguuaCaaggcagauucu..........................................................   1         1          seq
    ......................ucuuguCaaaaggcagauucu..........................................................   1         1          seq
    ......................ucuuguuaaaaggcagaCucu..........................................................   2         1          seq
    ......................Ccuuguuaaaaggcagauucu..........................................................   7         1          seq
    ......................ucuGguuaaaaggcagauucu..........................................................   1         1          seq
    ......................ucuuguuaaaaggcagauucA..........................................................   54        1          seq
    ......................ucuuguuaGaaggcagauucu..........................................................   1         1          seq
    ......................ucuuguuaaaaUgcagauucu..........................................................   1         1          seq
    ......................ucuuCuuaaaaggcagauucu..........................................................   2         1          seq
    ......................ucuuguuaaaaggUagauucu..........................................................   6         1          seq
    ......................ucuuguuaaaaggcagauucuU.........................................................   6         1          seq
    ......................ucuuguuaaaaggcagauucuA.........................................................   3         1          seq
    ......................ucuuguuaaaaggcagauucuAa........................................................   1         1          seq
    ......................ucuuguuaaaaggcagauucugau......................................................   1         0          seq
    .......................cuuguuaaaaggcagauucA..........................................................   1         1          seq
    .......................cuuguuaaaaggcagauucu..........................................................   4         0          seq
    .......................cuuguuaaaaggcagauucugau......................................................   2         0          seq
    .......................cuuguuaaaaggcagauucugauu.....................................................   1         0          seq
    .......................................uucugauucagggaccaagauuc.......................................   1         0          seq
    ..........................................................auucugcauuuuuagcaaguu......................   10        0          seq
    ..........................................................auucugcauuuuuagcaaguuU.....................   12        1          seq
    ..........................................................auucugcauuuuuagcaaguuc.....................   19        0          seq
```
